# Early development of synchrony in cortical activations in the human

**DOI:** 10.1016/j.neuroscience.2016.02.017

**Published:** 2016-05-13

**Authors:** N. Koolen, A. Dereymaeker, O. Räsänen, K. Jansen, J. Vervisch, V. Matic, G. Naulaers, M. De Vos, S. Van Huffel, S. Vanhatalo

**Affiliations:** a Division STADIUS, Department of Electrical Engineering (ESAT), University of Leuven, Leuven, Belgium; b iMinds-KU Leuven Medical IT Department, Leuven, Belgium; c Department of Development and Regeneration, Neonatology, University of Leuven, Leuven, Belgium; d Department of Signal Processing and Acoustics, Aalto University, Espoo, Finland; e Institute of Biomedical Engineering, Department of Engineering Science, University of Oxford, Oxford, UK; f Department of Children’s Clinical Neurophysiology, HUS Medical Imaging Center and Children’s Hospital, Helsinki University Central Hospital and University of Helsinki, Helsinki, Finland

**Keywords:** AC, Algebraic connectivity, ASI, activation synchrony index, cx-cx, cortico-cortical, EDTF, energy weighted temporal dependency function, GS, Global Synchrony, IHS, interhemispheric synchrony, iqr, interquartile range, IVH, intraventricular hemorrhage, MST, Minimum spanning tree, PMA, postmenstrual age, SAT, spontaneous activity transients, neonatal EEG, brain connectivity, biomarker, early development, brain monitoring

## Abstract

Early intermittent cortical activity is thought to play a crucial role in the growth of neuronal network development, and large scale brain networks are known to provide the basis for higher brain functions. Yet, the early development of the large scale synchrony in cortical activations is unknown. Here, we tested the hypothesis that the early intermittent cortical activations seen in the human scalp EEG show a clear developmental course during the last trimester of pregnancy, the period of intensive growth of cortico-cortical connections. We recorded scalp EEG from altogether 22 premature infants at post-menstrual age between 30 and 44 weeks, and the early cortical synchrony was quantified using recently introduced activation synchrony index (ASI). The developmental correlations of ASI were computed for individual EEG signals as well as anatomically and mathematically defined spatial subgroups. We report two main findings. First, we observed a robust and statistically significant increase in ASI in all cortical areas. Second, there were significant spatial gradients in the synchrony in fronto-occipital and left-to-right directions. These findings provide evidence that early cortical activity is increasingly synchronized across the neocortex. The ASI-based metrics introduced in our work allow direct translational comparison to *in vivo* animal models, as well as hold promise for implementation as a functional developmental biomarker in future research on human neonates.

## Highlights

• We study the early development of cortical activations synchrony index (ASI).
• Cortical activations become increasingly synchronized during the last trimester.
• Interhemispheric synchrony increases more than intrahemispheric synchrony.
• Our EEG metric ASI can be directly translated to experimental animal studies.
• ASI holds promise as an early functional biomarker of brain networks.

## Introduction

Large-scale spatio-temporal correlations in neuronal activity are considered to provide the functional basis for a range of brain functions in distributed networks (Bressler and Menon, 2010, Uhlhaas et al., 2010, Palva and Palva, 2011). These correlations are readily observed in the neuronal activity, as well as in the fluctuation of cerebral blood flow (Biswal et al., 1995, Jerbi et al., 2010), and they also correlate with behavioral states (Raichle, 2010, Palva and Palva, 2011, Hutchison et al., 2013).

Little is known about the early ontogenesis of functional communication in the human neuronal networks. Recent anatomical studies have disclosed an account of the microscopic development of structural networks in the human fetus (Kostovic and Jovanov-Milosevic, 2006, Kostovic and Judas, 2010), which sets the physical frame to how the early neuronal dynamics may emerge during latter half of gestation. Some features of large-scale spatial coordination in the electrical activity of the brain have been reported in sleeping human newborns (Tokariev et al., 2012, Omidvarnia et al., 2014). It is known that two modes of brain activity (Vanhatalo and Kaila, 2006) alternate in sub-second time scales between a relative quiescence and its interruptions by spontaneous activity transients (SAT, a.k.a. burst; Vanhatalo et al., 2005). These SATs are thought to provide the endogenous driver needed for activity-dependent wiring of the early brain networks, prior to onset of genuine sensory experience (Hanganu-Opatz, 2010, Kilb et al., 2011, Colonnese and Khazipov, 2012).

Early clinical studies on neonatal EEG established that the temporal co-incidence of these activity bursts between the hemispheres, commonly called “interhemispheric synchrony” (IHS), is a good marker of normally developing EEG activity at term age. A developmental increase in IHS was found during the last trimester of pregnancy (Lombroso, 1979), however, the existing literature is based on subjective and largely qualitative EEG assessment that compromises the validity of detailed findings. Moreover, there are no reports on spatial differences in the development of synchrony between cortical areas, yet histological studies have clearly established distinct developmental trajectories in the growth of long-range cortico-cortical (cx-cx) pathways (Judas et al., 2005, Kostovic and Jovanov-Milosevic, 2006).

We have recently developed and validated a measure for IHS, called activation synchrony index (ASI), which statistically quantifies the temporal coincidence of SAT in the cortical activity (Räsänen et al., 2013, Koolen et al., 2014b). Since late 1970s, the clinical EEG review has involved visual assessment of IHS as one of the key parameters of EEG maturation and normality (Lombroso, 1979). Our benchmarking study with visual reading and other synchrony measures showed that ASI is most accurately emulating the clinically recognized phenomenon of cx-cx EEG synchrony. This has opened the possibility to study how the synchrony between cortical activations evolves during prematurity. In the present study, we aimed to characterize the developmental correlations of cx-cx synchrony during the last ten weeks of pregnancy, which is characterized by the rapid development of long-range cx-cx connections. In particular, we wanted to disclose potential spatial gradients, as well as assess whether the developmental changes are robust enough to even allow using the ASI-based cx-cx synchrony as a maturational measure.

## Experimental procedures

### Data acquisition

The main dataset consisted of 22 recordings in 20 infants, recorded at a postmenstrual age (PMA) of 30–44 weeks at the Neonatal Intensive Care Unit of the University Hospitals of Leuven, Belgium (Koolen et al., 2014a, Koolen et al., 2014b). In this pilot-study, we used broad inclusion criteria and have included 2 infants with intraventricular hemorrhage (IVH) grade III, however no infants had parenchymal lesions such as parenchymal infarction or periventricular leukomalacia. Most importantly, the infants were clinically stable by the time of EEG recording. More clinical details of the infant group are given in Table 1. Two infants had consecutive recordings performed for clinical reasons to assess their brain development. Their two EEG recordings were entered as independent samples, however we also computed the group results with only one recording from each infant, and we saw no meaningful differences in the results. The lower age limit was set to 30 weeks PMA, to assess the developmental window at an age when the majority of thalamocortical connections is already established, whereas an intensive growth of cortical–cortical connections exists (Kostovic and Jovanov-Milosevic, 2006, Jovanov-Milošević et al., 2009, Kostovic and Judas, 2010). One term infant was excluded because of missing tracé discontinue EEG patterns, the foundation of ASI analysis. The recording time was at minimum 4 h, and a clinical expert (A.D.) selected the most discontinuous EEG, resembling quiet sleep in older patients, for 2 × 10 min in each recording. All EEG measurements were recorded at 250 Hz, with 8 electrodes (Fp1, Fp2, C3, C4, T3, T4, O1, O2) placed according to the 10–20 standard locations and reference electrode Cz (BRAIN RT, OSG equipment, Mechelen, Belgium). The protocol was approved by the Ethics Committee of the University Hospitals of Leuven, Belgium. Preprocessing the data involved applying a 50 and 100 Hz Notch filter and a 1–20-Hz band pass filter to capture the interesting burst information present in this frequency range.

### Analysis of synchrony

We analyzed the synchrony between cortical areas by using the recently developed measure ASI, which estimates the temporal relationships between newborn cortical events (for further details, see Räsänen et al., 2013). Our primary aim was to study the development of ASI from prematurity to term age. In addition, we also studied spatial differences in ASI development, as well as the temporal fluctuations of ASI within each recording session in order to assess its methodological stability and potential use as a maturational measure of functional connectivity.

Technically, ASI (and mutual information in general) captures all non-linear correlations between the signals and is invariant to any coordinate transforms of the input data, which is not true for normal correlational analysis (see, e.g., Herzel and Große, 1995). We have directly compared ASI to the cross-correlation of the raw signal or the signal energy function, neither of which is suitable for capturing the temporal relations of interest in our context (Räsänen et al., 2013). Other somewhat comparable measures would include the correlation coefficient between amplitude envelopes (see e.g. Hipp et al., 2012, Tokariev et al., 2015b) and the computation of the slope between quantized amplitude envelopes (e.g. Omidvarnia et al., 2014, Omidvarnia et al., 2015), neither of which is designed to statistically test the presence of temporal delays, a key feature of interest in assessing cx-cx activation synchrony in the preterm EEG signal. Due to the computational robustness and the ability to emulate clinical EEG review, ASI is also a putative, practical biomarker to describe preterm maturation in the context of a real-world hospital environment.

### Computation of ASI

Temporal relationships between pairs of EEG signals were computed using a previously described measure ASI (Räsänen et al., 2013), which was recently shown to perform well in distinguishing normality in EEG traces from term patients (Koolen et al., 2014b). ASI provides a statistical measure for the temporal coupling of two quantized EEG amplitudes with higher ASI values reflecting larger synchrony between the signals, whereas an ASI of zero means that the two signals are statistically independent. The algorithm consists of the following steps (Räsänen et al., 2013):

1. The signal is first down sampled to 50 Hz. Higher frequencies (which are related to the burst events) are pre-emphasized with a first order FIR high-pass filter (H(z) = 1–0.95 z^−1^).
2. Amplitude envelopes of the pre-processed signals are obtained by computing the fast Fourier transformation (FFT) using a sliding Hamming window of 2 s and a step size of 100 ms (following the optimization performed in Räsänen et al., 2013), and then summing up the amplitudes of the frequency bins in the relevant frequency range of 1.5–20 Hz for each 100-ms frame.
3. The signal envelopes are quantized into *Q* discrete amplitude levels (or “*states*”) by clustering a random subset of the samples with the standard k-means algorithm and then assigning each sample to the nearest resulting cluster. As a result, two discrete sequences corresponding to the two input signals *A* and *B* are obtained
  $$X_{A}=a_{1}\text{,}a_{2}\text{,}\ldots \text{,}a_{N}\;\text{and}\;X_{B}=b_{1}\text{,}b_{2}\text{,}\ldots \text{,}b_{N}\;(\text{with}\;a_{i}\text{,}b_{i}\in [1\text{,}2\text{,}\ldots \text{,}Q])$$
  The discrete representation enables the estimation of the joint probabilities of the quantized amplitude values across the two signals. In the present work, *Q* = 8 quantization bins were used similarly to Räsänen et al. (2013) and Koolen et al. (2014b).
4. In the next step, a so-called energy weighted temporal dependency function (EDTF) between the two quantized envelopes is calculated. EDTF is a mutual information-based metric that measures the overall (non-logarithmic) statistical dependencies across all possible discrete signal state pairs at different relative temporal lags between the signals of interest, weighting the degree of statistical coupling of each state pair by the amount of signal energy associated with these states. More specifically, ETDF is calculated using the following formula:
  (1) $$\text{ETDF}(\tau )=\underset{a\text{,}b}{\sum }\text{AMP}(a)\text{AMP}(b)\frac{{\text{P}}_{\tau }{\left(a\text{,}b\right)}^{2}}{\text{P}(a)\text{P}(b)}$$
  where P*τ* (*a*,*b*) denotes the probability of observing level *a* in the first channel and level *b* in the second channel when the second signal is delayed by *τ* seconds relative to the first signal, and where *τ* = [−5, 5] s was used in the present work. The resulting ETDF is offset-normalized to have a minimum-value of zero within this ±5-s range, assuming that the true coupling between the signals should be zero at larger temporal delays and thereby compensating for any estimation biases in the mutual information that could result from the use of finite data.
5. Finally, the ASI is obtained by calculating the ratio of the normalized EDTF value at zero time lag (*τ* = 0) over the mean value of the normalized ETDF function over the 10-s range (*τ* ∊ [−5,5] s), effectively reflecting the magnitude of the zero-delay coupling between the two signals in comparison to delayed or temporally smeared concurrent activation in the signals.

For the present study, ASI was initially computed for EEG epochs (or ASI windows) of 1 min and 2.5 min. It was originally shown (Räsänen et al., 2013), that ASI is more stable with longer (>2 min) window lengths. However, we have later shown that averaging over shorter windows may be preferred (Koolen et al., 2014b), perhaps due to limited long-range temporal correlations in the newborn EEG. Using multiple shorter windows is also better suited for the analysis of older neonates with shorter quiet sleep epochs. As a compromise of the above considerations, we studied the ASI stability (see below) using the median ASI value over all 1 min and 2.5 min EEG epochs as the representative ASI measure for the given subject. Additional analyses were performed for ASI values of each single EEG epoch.

### Assessment of ASI stability

**Fig. 1:**
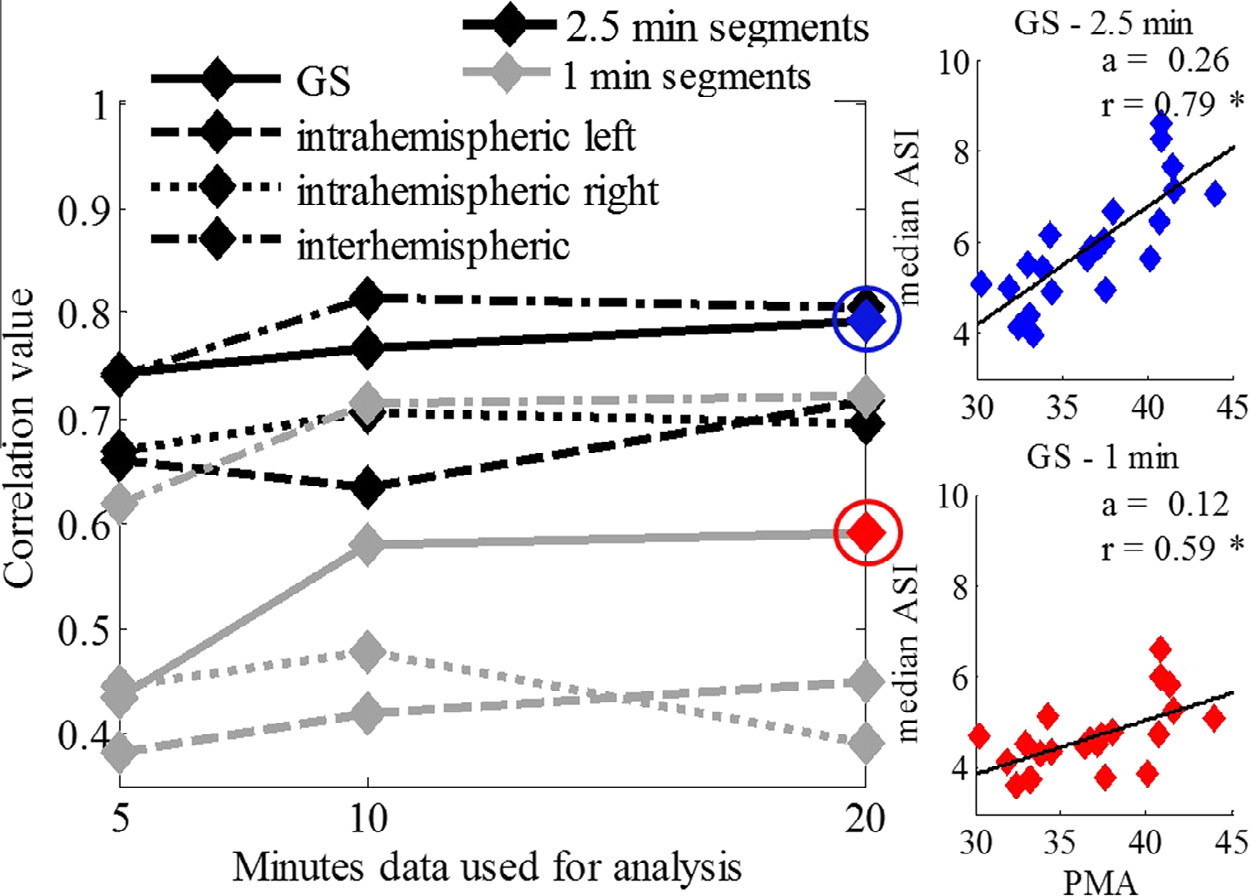
Comparison of ASI analysis settings with respect to developmental correlations. Left side graph shows correlation coefficient (*r*) between ASI and PMA in the same dataset when ASI is computed using different amount of data (*x* axis), different analysis windows (1 min vs 2.5 min), or different combinations of channels. A little increase in *r*-values is found when using longer EEG epochs. On the right side, PMA correlations are shown for 1 min and 2.5 min ASI windows. Both correlations are significant, however use of 2.5 min ASI windows gave clearly steeper developmental trends and higher correlation values.

The underlying general assumption in IHS is temporal stability, hence ASI would be ideally expected to yield relatively stable values regardless of the data length used for its computation. In order to test whether ASI estimates are really so stable, we first analyzed how the length of EEG epoch would affect the findings, and whether the choice of the ASI analysis window influences the observed correlations to the PMA. We found that a significant developmental increase in the ASI is seen for all tested amounts of EEG data, however, there was a slight increase in correlation coefficients when more EEG data was used (Fig. 1). In addition, increasing the ASI window length from 1 min to 2.5 min resulted in ASI vs PMA correlations with both stronger correlation coefficients and steeper slopes (1 min: *r* = 0.59, slope *a* = 0.12; 2.5 min: *r* = 0.79, slope *a* = 0.26) (Fig. 1). Based on these observations, all later analysis was performed using 2.5 min ASI windows, instead of 1 min windows.

### Spatial analysis of ASI

We computed ASI estimates between all monopolar channel combinations. In addition, ASI estimates for bipolar derivations in both hemispheres were obtained for Fp-C, Fp-T, Fp-O, C-O, T-O, C-T. Then, we formed the following groups of signal pairs to study spatially specific developmental correlations: (i) Global Synchrony (GS) was computed as the average of all 28 pairwise ASI values to characterize global connectivity, (ii) Interhemispheric synchrony was computed as the average of symmetric channel combinations between hemispheres, (iii) Intrahemispheric Synchrony was computed by taking the average of all six channel combinations in each hemisphere (for the left hemisphere: Fp1-C3, Fp1-T3, Fp1-O1, C3-O1, T3-O1 and C3-T3; for the right hemisphere Fp2-C4, Fp2-T4, Fp2-O2, C4-O2, T4-O2 and C4-T4), (iv) Synchrony in the anterior and posterior areas were computed as the average of all 8 channel combinations in the respective areas. A scheme of these spatial subgroups is shown in Fig. 3. In addition, we assessed another possibility to reduce the number of pairwise ASI estimates by extracting the first component with the principal component analysis.

### Network measures

The pairwise connectivity matrix reflects interactions in the network that can also be quantified using graph metrics. Within these metrics, each individual EEG signal can be considered as the node, the signal pair is the edge, and the connectivity measure, here ASI, can be considered the weight of this edge. The commonly used graph theoretical metrics (Bullmore and Sporns, 2009, Stam and van Straaten, 2012) may have limited utility in very sparse graphs, or graphs with highly varying edge levels (see for more details Stam et al., 2014), which characterize our present situation. Hence, we decided to quantify the global network properties with two alternative graph measures: the Minimum spanning tree (MST) and the Algebraic connectivity (AC).

The MST is an acyclic sub-network between nodes (here, EEG signals) that allows a quantitative assessment and comparison of networks with presumably low bias as compared to more traditional graph metrics (Stam et al., 2014, Tewarie et al., 2015). We computed the metric MST mean using the freely available program BrainWave (http://home.kpn.nl/stam7883/brainwave.html; Stam et al., 2014). This returns the mean value of all edges selected for the MST of the given individual recording. In contrast to the mean over all 28 pairwise channel connections (measure GS above), MST mean will only take a subset of the strongest connections, which may reduce sensitivity to random variability.

AC is a metric often used to quantify the connectivity; a low AC means that its cost to cut the connectivity graph in approximately two parts is low (cfr. smaller weights between nodes). For maturing babies, edges between the brain regions would be expected to become stronger, leading to more costly graph cuts and, consequently, higher AC values. AC is obtained as the second-smallest eigenvalue of the Laplacian matrix *L* of the original connectivity matrix, and it is strictly positive (under the assumption of a connected graph) and increasing if more links are added to *L*. (Bertrand and Moonen, 2013).

## Results

### Intraindividual stability

**Fig. 2:**
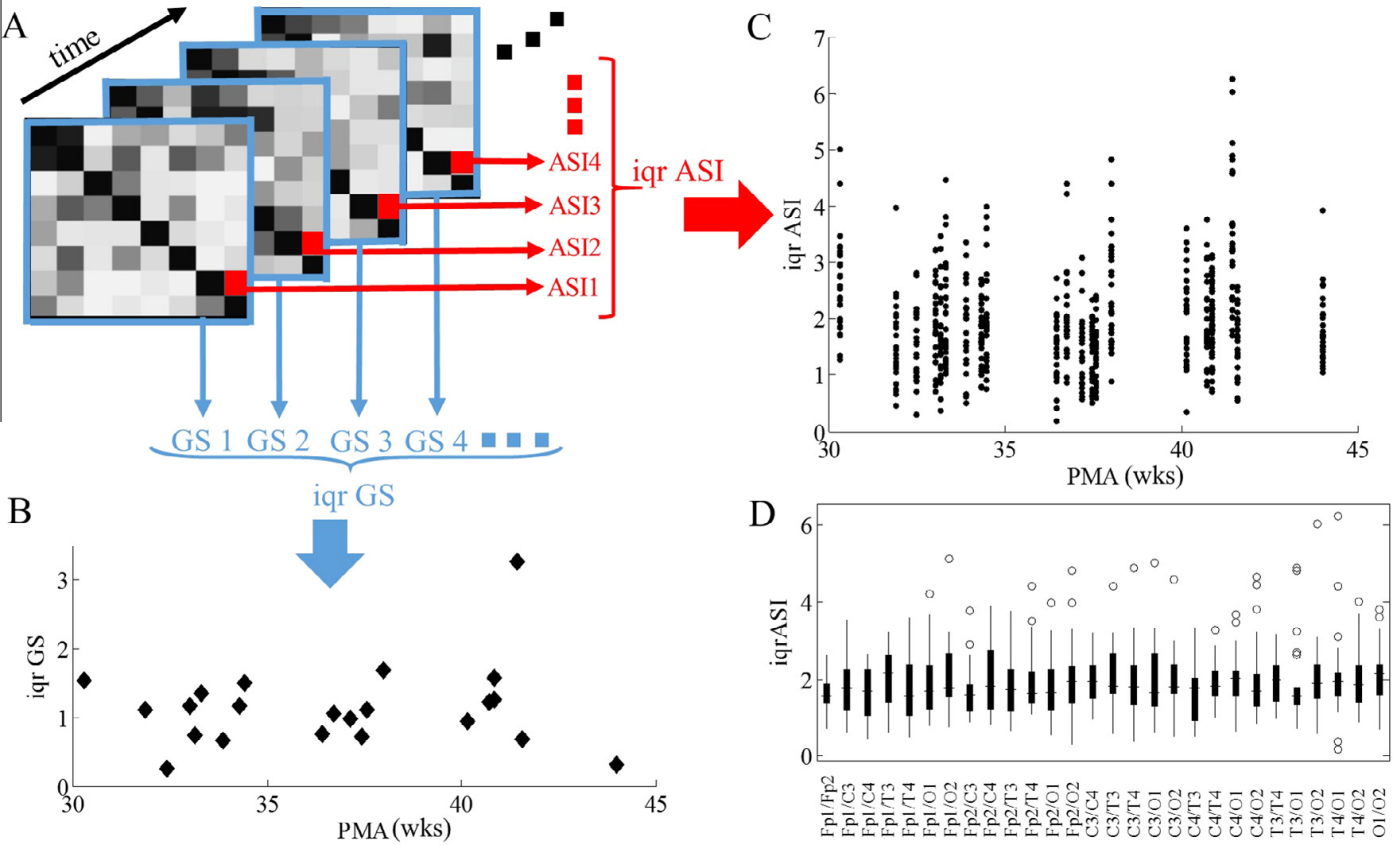
Intraindividual ASI stability and its development. (A) Synchrony matrices derived from subsequent EEG epochs of 2.5 min including all 28 ASI values derived from the possible channel pair combinations, (B) temporal variability of global synchrony values defined as the iqr of 8 global synchrony values (from the subsequent synchrony matrices) (see also Fig.3A), (C) spatial variability derived from the 28 channel combinations as the iqr of each specific ASI over consecutive epochs, calculated for each individual without significant developmental trend (in function of PMA), (D) similar interquartile variability over all patients for each single channel pair combination.

We first examined the temporal and spatial variability of ASI by computing the interquartile range (iqr) of all ASI values within each connectivity matrix (spatial variability), and by computing the iqr of the GS in successive ASI windows (temporal variability, Fig.2B). Our findings show that spatial variability is large (Fig.2C), in the order of 1 to 6 ASI units within an individual, but without a significant developmental trend. There were also no significant differences between specific channel combinations (Anova test: 0.96; Fig.2D). Temporal variability of the GS was expectedly lower, in the order of one ASI unit, and it showed no significant developmental change (Fig.2B). These findings suggest that the search of developmental correlations is likely more reliable after spatial averaging over regional groups.

### Spatial ASI analysis

**Fig. 3:**
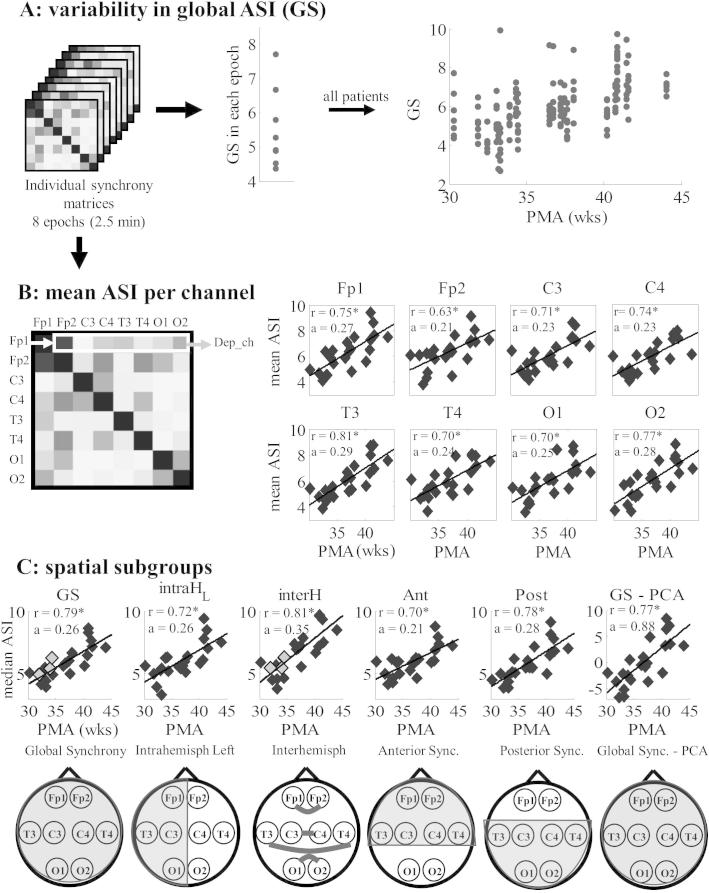
Spatial ASI analysis and its development. (A) In addition to the temporal variability seen as the interquartile range of GS values in successive epochs, there was also an overall increase in GS values with increasing PMA, (B) graphs depicting the developmental change in the mean ASI of each EEG channel compared to the other 7 channels, which have all significant correlations, (C) graphs representing the developmental change in the mean ASI over the given spatial subgroup as schematically shown in the topoplots. Premature infants with early IVH grade III are shown in gray (for GS and interhemispheric synchrony). The right most plot depicts developmental change of the first component of principal component analysis (PCA). Significance of the correlation is depicted with an asterisk after correlation coefficient ‘*r*’. The value ‘*a*’ depicts the slope of linear regression computed for the given graph.

We observed a clear overall increase in GS values over the course of early development (Fig.3A). To see whether this was systematically related to specific EEG signal combinations, we assessed the mean ASI of each EEG signal compared to the other seven EEG signals. As shown in Fig.3B, each of the eight EEG signals showed a significant developmental increase in their mean connectivity, and there were relatively minor differences between individual channels in the slopes of ASI vs. PMA.

The above findings together suggested that the development of activation synchrony is global, and a spatial combination across wider channel groups is possible. We hence computed the median ASI across spatial groups (Fig.3C), and we found significant developmental correlations for all groups. There were, however, clear differences between spatial groups: the interhemispheric ASI was highest (*r* = 0.81, slope *a* = 0.35), followed by the GS (*r* = 0.79, *a* = 0.26), while the intrahemispheric ASI was the lowest (*r* = 0.72, *a* = 0.26 and *r* = 0.69, *a* = 0.18 for left and right hemispheres, respectively). The ASI values of the two premature infants with early IVH grade III were in line with the others (Fig.3C, patients labeled in gray for GS and interhemispheric ASI).

### Global metrics of ASI-based connectivity

**Fig. 4:**
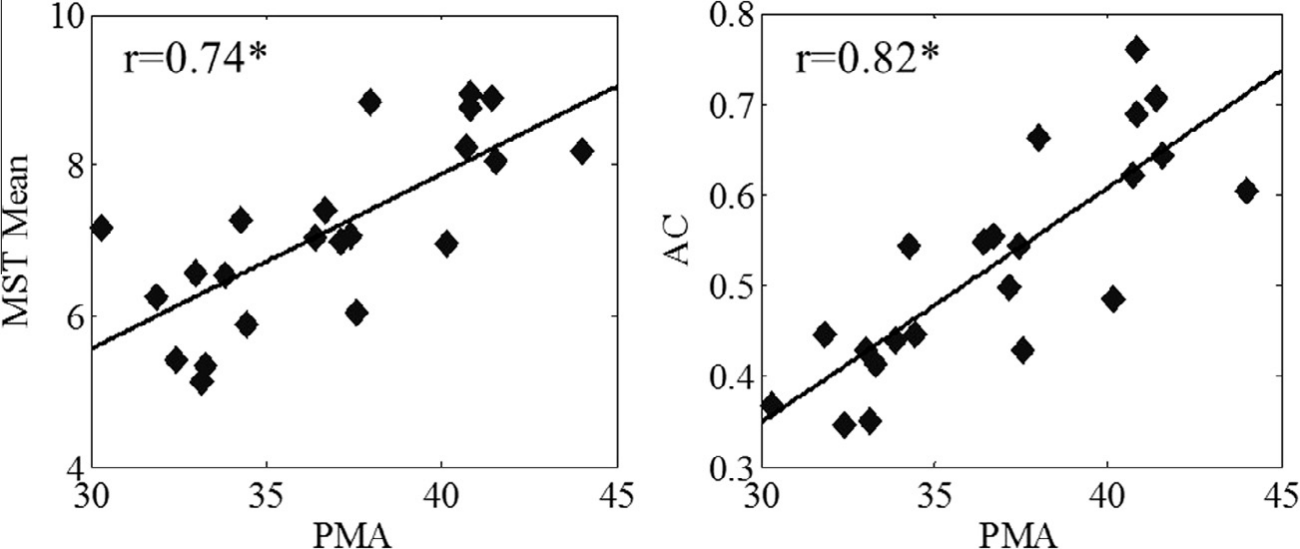
Developmental change of graph metrics, MST mean and algebraic connectivity, both of which showed a significant correlation with PMA.

We first tested reduction of analytic dimensions and spatial variability by using the principal component analysis. Its first component was found to have a highly significant correlation (*r* = 0.77, *p* = 2.62 * 10^−5^), as well as a steep relationship to PMA (slope *a* = 0.88), suggesting that global properties of the connectivity might also reveal robust developmental correlations. Indeed, both measures of global graphs, the mean MST and AC, were found to correlate significantly to PMA (mean MST: *r* = 0.74, *p* = 7.2 * 10^−5^; AC: *r* = 0.82, *p* = 3.3 * 10^−6^) (Fig. 4).

Finally, we examined the mutual correlations between the three global measures – GS, mean MST and AC – which all had shown significant developmental changes. As expected, we observed high correlations for respectively MST mean–AC, MST mean–GS, GS–AC: *r* = 0.93, *p* = 5.2 * 10^−10^ and *r* = 0.94, *p* = 1 * 10^−10^ and *r* = 0.94, *p* = 3.8 * 10^−11^.

### ASI in bipolar derivations

**Fig. 5:**
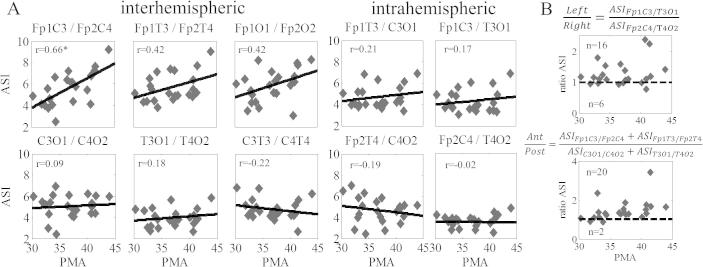
ASI in bipolar derivations. (A) Developmental changes in ASI computed from bipolar derivations for both interhemispheric and intrahemispheric channel combinations. Note that the correlation is often not significant and its strength (*r*) is smaller as compared to monopolar derivations (Fig. 3). (B) Hemispheric and anterior–posterior comparisons reveal significant asymmetries. Comparison of frontal and posterior interhemispheric connections shows frontal dominance in 20 out of 22 cases, while the left side shows stronger ASI in 16 out of 22 cases.

In order to provide an additional, clinically familiar benchmark, we computed ASI between bipolar signals, the preferred montage in the clinical EEG reading (André et al., 2010). We have shown earlier that different bipolar derivations are not directly comparable in terms of their temporal stability (Koolen et al., 2014b) or sensitivity to therapeutic maneuvers (Vanhatalo, unpublished observations), hence they may also exhibit different developmental correlations. We found that frontal interhemispheric connections are stronger correlated to PMA compared to posterior brain regions (Fig. 5). Indeed, no significant correlations are found between PMA and bipolar derivations in the posterior region or in the intrahemispheric channel combinations (*r* < 0.3 in all cases).

Finally, we wanted to see if there is a systematic spatial asymmetry in the bipolar derivations. Intraindividual comparison of anterior and posterior interhemispheric ASI showed that 20 out of 22 infants had higher ASI values in their anterior areas, which was statistically highly significant (*p* < 6 * 10^−5^; binomial statistics). In addition, intraindividual comparisons of left and right intrahemispheric derivations showed that 16 out of 22 infants had higher ASI values on their left hemisphere, which was statistically significant (*p* < 0.03; binomial statistics).

## Discussion

We show that the synchrony between cortical activations strongly correlates with development during the last two months before normal birth in the human. The present quantitative findings are fully compatible with the earlier, mainly qualitative visual EEG observations (Lombroso, 1979). Our work extends prior knowledge by presenting how the developmental change is global, and it reflects the recently shown histological maturation of the corresponding cx-cx networks. It is conceivable that the pace of early functional brain maturation may differ between infants that normally develop *in utero* vs. those that are born very prematurely and hence undergo longer *ex utero* development. The effects of *ex utero* experience on EEG maturation are, however, so small (Shany et al., 2014), that they likely fall within the normal inter-individual variability and the limits of analytical accuracy in our work. A prudent study on the relative contributions of these effects, including various clinical factors, will need larger prospective data collection with repeated EEG measures from each individual to minimize the effects of physiological interindividual variability.

While the age range in this study only extends to term age, the long-range network organization is known to develop many more years into adolescence (Uhlhaas et al., 2010). Studying these later developmental trajectories of brain connectivity will need different analytical methodology, because ASI is only applicable to measure temporal synchrony between intermittent cortical activities, the hallmark of neonatal EEG activity (Vanhatalo and Kaila, 2006, André et al., 2010, Räsänen et al., 2013).

Comparison of brain regions showed that the ASI-based connectivity develops in a global manner, with relatively minor differences between cortical areas. This was somewhat surprising, given the spatial gradients in the development of structural long range cx-cx connections (Judas et al., 2005, Jovanov-Milošević et al., 2014). Our spatial findings cannot be directly compared to prior studies, because cortical activations comparable to ASI have been studied in central areas only (Meyerson, 1968, Lombroso, 1979, Marcano-Reik et al., 2010). However, recent work on spatial amplitude relationships suggested that the high cortical activity mode shows developmental gradients and an emergence of frontal and posterior groups toward late gestation (Omidvarnia et al., 2014). Our present findings show an anterior-posterior gradient in ASI without apparent developmental trajectory. This is compatible with the idea that stronger anterior IHS cx-cx correlations may reflect more global functional connectivity in the precentral areas as compared to the more spatially segregated postcentral areas that consist of sensory and association cortices.

Our group level hemispheric comparison revealed a significant asymmetry with left hemisphere showing relatively higher ASI levels in a majority of infants. This ASI asymmetry was computed between two bipolar derivations within the given hemisphere, so it reflects hemisphere level temporal coordination of spontaneous activations. Prior studies have consistently shown that functional and structural hemispheric lateralization begins very early in development (reviewed by Behrmann and Plaut, 2015), however we are not aware of prior EEG studies showing hemispheric differences in connectivity measures in human preterm infants. We find it reasonable to speculate that the ASI asymmetry found in our work reflects the recently reported relative advance in the left side structural connectivity (Ratnarajah et al., 2013).

In addition to spatial averaging of ASI over channel combinations, we showed that graph metrics may disclose similar, significant developmental correlations. However, the limited number of (eight) electrodes available in our dataset did not allow a pertinent assessment of graph measures at the hemispheric or lobar level. Yet, the observations support prior studies (Omidvarnia et al., 2014, Omidvarnia et al., 2015) in a way that these graph measures may provide a useful tool for the developmental indexing of functional connectivity. Future studies with many more EEG channels for pertinent developmental graph analysis are warranted.

While our findings are readily explained in the context of other physiological and anatomical literature, there are some technical considerations that limit the quantitative accuracy of our results. First, the spatial comparisons may be influenced by the number of electrodes and montages. The present dataset consists of eight channel recordings, which is the standard clinical practice (André et al., 2010), but limited with respect to spatial resolution available in the neonatal scalp EEG (Grieve et al., 2004, Odabaee et al., 2014, Tokariev et al., 2015a). We computed most spatial analysis with monopolar montage (Cz reference), which is not fully neutral but likely the best compromise. This may bias findings toward less differences between electrodes, which is likely given the findings from our further assessment with bipolar derivations that showed spatial gradients in both anterior-posterior and left-right direction. The interelectrode distance between monopolar reference and the ‘recording electrode’ should not have much influence, because of the high spatial specificity shown in the newborn scalp EEG (Odabaee et al., 2013, Odabaee et al., 2014). Second, more EEG data available for the ASI analysis per patient could reduce the amount of random temporal variability. The amount of high quality EEG data available from human newborn infants is always limited, and our prior work (Koolen et al., 2014b) has shown 5–10 min of EEG yielding reasonably stable values. The possible bias from such technical variability would lead to the underestimation of correlations between ASI and development, which was already found to be highly significant with this data.

In addition to the physiological implications, our work does also suggest that ASI-based quantitation of functional cortical synchrony might offer a useful developmental measure in clinical studies. The recordings used in our present work are available in all medical centers doing routine neurophysiological service for neonates, and we demonstrate here the optimized analysis settings for such datasets. This opens a novel possibility to perform retrospective studies of brain functional connectivity in any developmental disorders that were recorded for clinical reasons during neonatal period. A particular extension of clinical research interest is the possibility to construct developmental indices from the ASI measures. Our work reports many ASI-based measures holding promise as a feature in developmental growth charts, which can be constructed using a well characterized, prospectively collected control population. Such growth charts would provide functional biomarkers that are very much needed in future studies on early development or in association with therapeutic interventions.

## Figures and Tables

**Table 1:** Overview patient data set: GA (gestational age), birth weight (in g), gender (girl/boy), PMA (postmenstrual age), cranial ultrasound (with IVH: intraventricular hemorrhage) and patient’s outcome (BSID: Bayley Scales of Infant Development)

Pt	GA	Birth weight	Gender	PMA	Cranial ultrasound	Outcome: abnormal if BSID-II MDI or PDI < 70
1_1	24	700	G	31.86	IVH-III/inhomogenic	BSID-II (24 mo) MDI 55 PDI 88
1_2				33.86	Hyperechogenicities > 2w	
2	31.86	1900	B	33.14	Normal cranial ultrasound	BSID-II (24 mo) MDI 66 PDI 74
3_1	28	1390	B	30.29	IVH-I unilateral	Normal outcome (24 mo)
3_2				32.43		
4	32.86	1900	B	33	Normal cranial ultrasound	Normal outcome (24 mo)
5	27.71	920	B	34.29	IVH-III	Normal outcome (24 mo)
6	31.14	800	G	34.43	IVH-I bilateral	Normal outcome (9 mo)
7	31.57	2200	G	33.29	Normal cranial ultrasound	Normal outcome (9 mo)
8	40	3330	G	44	Normal cranial ultrasound	Normal outcome (9 mo)
9	40	3355	B	40.86	Normal cranial ultrasound	Lost to follow up
10	30.29	1385	G	36.71	Normal cranial ultrasound	Normal outcome (9 mo)
11	37	2970	G	38	Dural sinus malformation	Normal outcome (9 mo)
12	27	1160	B	37.43	Normal cranial ultrasound	Lost to follow up
13	30.86	1300	G	37.14	Normal cranial ultrasound	Normal outcome (9 mo)
14	25.71	720	B	40.71	Normal cranial ultrasound	Normal outcome (9 mo)
15	25.71	900	B	40.14	Normal cranial ultrasound	Normal outcome (9 mo)
16	39.14	2820	B	41.43	Aqueductal stenosis	Normal outcome (9 mo)
17	40.86	4200	G	40.86	Normal cranial ultrasound	Normal outcome (9 mo)
18	32	1800	B	37.57	Normal cranial ultrasound	Lost to follow up
19	32.86	2040	B	36.43	Normal cranial ultrasound	Normal outcome (9 mo)
20	26	940	B	41.86	Normal cranial ultrasound	BSID-II (9 mo) MDI 68 PDI 82
